# Supplementary material for: The identity blueprint: Decoding the professional identity of Basic Sciences Medical Teachers in Pakistan by developing and pilot testing a questionnaire
Source: PLoS One. 2026 Feb 6;21(2):e0340290. doi: 10.1371/journal.pone.0340290 (PMC12880675; doi:10.1371/journal.pone.0340290)
Supplement: S1 Appendix — (PDF) [file pone.0340290.s001.pdf]

Scale to measure Professional identity formation of Basic Sciences Medical Teachers

This scale has been designed to evaluate factors forming professional identity of Basic Sciences Medical Teachers. Identity formation involves understanding oneself in relation to others and the world at large. Professional Identity (PI) in the medical profession is understood as how a doctor thinks of himself as a doctor. We would define PI as “It is the feeling of belonging to the basic medical sciences teaching profession and to a larger body of basic sciences medical teachers.

1 strongly disagree, 2 disagree, 3 neutral, 4 agree, 5 strongly agree

No	Items	1	2	3	4	5
1	I wish I could rewind the time and chose clinical field					
2	I feel proud to be a medical teacher					
3	It syncs well with family life					
4	My seniors encourage me grow professionally					
5	I wish I could practice as a part-time physician					
6	My hard work is usually acknowledged at workplace					
7	I have started accepting people the way they are					
8	I stay calm in stressful situations					
9	I take my students’ words as personal evaluation of myself					
10	I keep myself updated with the latest trends in my field					
11	I foresee myself in a better position in my career					
12	I am glad I bring positive changes in society					
13	The only reward I get at workplace is, more work!					
14	I am positively challenged at workplace					
15	Teaching comes naturally to me					
16	I feel accomplished when I see my students progressing					
17	People think medical teachers are not doctors					
18	I consider my profession a legacy (sadqaejaariah)					
19	I enjoy liberty to exercise innovative ideas at workplace					
20	I frequently attend workshops, conferences and seminars					
